# A Comparative Study of Pectin Green Extraction Methods from Apple Waste: Characterization and Functional Properties

**DOI:** 10.1155/2022/2865921

**Published:** 2022-12-19

**Authors:** Marwa Hanafy Mahmoud, Ferial Mohamed Abu-Salem, Dina El-Sayed Helmy Azab

**Affiliations:** Department of Food Tech., National Research Centre, Dokki, Cairo, Egypt P.O. Box 12622

## Abstract

Traditional methods of pectin extraction led to drop quality, yield, functional properties, and excessive time. The objective of our research is to produce high-quality pectin from apple pomace as food processing by-product. Four nonconventional methods of extraction (microwave, ultrasound, citric acid, and organic acid mixture (citric acid, ascorbic acid, and acetic acid)) were compared to conventional extraction of pectin in terms of yields, thermal behavior, functional groups, antioxidant activity, and functional properties. Citric acid extraction method gave the highest yield (22%) compared to other methods. The extraction of pectin by organic acid mixture maximized the galacturonic acid index to 87.58%;. Also, it was changed from structural into: compacted, multilaminated, and flaky surface compared to the other samples (more porous and hollow opening structural) as well as increased stability of pectin particles in colloids as a result of increasing the charge on particles to -59.42, beside its higher thermal stability of pectin behaviors, which reflected on improving all functional properties compared to the other methods. On the other side, microwave-extracted pectin had the highest antioxidant activity (3-4 times) compared to other extraction methods. In conclusion, extraction using organic acids, microwave, and ultrasonic led to improve the pectin quality and could be used in high-temperature food products, like bakery products.

## 1. Introduction

The major quantities of food waste in the world were about 30%, including 45% from vegetables and fruits, 26% from drinking industry, 21.3% from dairy processing, and 14.8% from the production of fruits and vegetables [1]. Citrus peel and apple pomace form the prime sources for commercial production of pectin [2]. Pectin contributes about two-thirds of plant cell wall structure. Pectin is a heterogeneous polysaccharide; the major is a polymer of linear chain covalently linked *α* (1-4) D-galacturonic acid and has a glycosidic bond between anhydrogalacturonic residues and methyl-esterified carboxyl groups. The usage of pectin is as a gelling agent in food products such as jellies, also known as texture stabilizing in fruit juices and acidified milk; as an antioxidant in fortified foods, spreads, ice cream, and meat products; and as a fat replacer in low-fat food products [3, 4]. The degree of esterification (DE) of pectin is classified into two categories: high-methoxyl pectin (HMP) 60-75% and low-methoxyl pectin (LMP) 20–40%.

Extraction methods of pectin strongly influence the yield and quality of pectin. Pectin extractions include single methods such as acid, alkali, and enzyme extraction and combined methods such as the use of microwave or ultrasound to improve the pectin characteristics. The conventional method for pectin extraction resulted in low pectin quality as a result of the length of time required for extraction. Therefore, microwave extraction (ME) and ultrasound extraction (UE) are new methods that were introduced in pectin extraction to increase the quality and yield of pectin [5, 6]. Green extraction using organic acids, ultrasound, microwave, supercritical fluid, and accelerated solvent extraction are novel methods that have been developed in use in efficient technology for the retrieval of value-based vital compounds from different plants [7]. Ultrasound irradiation extraction is a nonthermal process that applies ultrasound energy to cause cavitation phenomena, resulting in an increase in the release and diffusion of materials; ultrasound extraction achieved several advantages; they include high extraction efficiency and higher yields than conventional heating methods [8]. Microwave extraction has the ability to reduce the time, cost, and solvent consumption, and it has a higher rate of extraction. The microwave heat increased the mass transfer and induced a collapse in the pectin, cellulose, and hemicellulose networks; microwave energy enhances the solvent penetration in plant samples [9].

The physicochemical properties of pectin (including molecular weight, degree of methylation, and esterification) are related to the chemical structure of pectin (polysaccharide structure, protein moiety, and acetyl groups) which plays a major role in functional properties (such as viscosity, solubility, and gelling properties) [10]. The objectives of this research were to study the effect of different pectin extraction methods (conventional citric acid, ultrasonic, microwave, and organic acids) to improve its functional properties.

## 2. Materials and Methods

### 2.1. Material

Anna apple pomace was obtained after juice extraction using an electric grinder and oven air dried according to [11] at 50°C. All chemicals were analytical grade purchased from Sigma-Aldrich.

### 2.2. Methods

#### 2.2.1. Pectin Extraction

**Step 1 (extraction treatments).**

- Heat conventional (HC)

Apple pomace powder (100 grams) was mixed with 2 liters of distilled water with continuous stirring using mechanical stirrer (Framo-Geratetechnik, Model R20, Germany) at room temperature (25°C) for 1 hour. The pH was adjusted to 1.9 using 2 N HCl with continuous stirring for about 10 minutes. This mixture was boiled for 1 hour using hot plate (Nour, Model 2007, Egypt).

(b) Citric acid extraction (CI)

Apple pomace powder (100 grams) was mixed with 2 liters of distilled water until stirring. The pH was adjusted to 1.9 using 2 N citric acid with continuous stirring using mechanical stirrer using mechanical stirrer (Framo-Geratetechnik, Model R20, Germany) at room temperature (25°C) for about 10 minutes. This mixture was boiled for 1 hour using hot plate (Nour, Model 2007, Egypt).

(c) Organic acid mixture (ORG)

Apple pomace powder (100 grams) was mixed with 2 liters of distilled water until stirring. The pH was adjusted to 1.9 using mixture of organic acid mixture (citric acid, ascorbic acid, and acetic acid) with continuous stirring using mechanical stirrer (Framo-Geratetechnik, Model R20, Germany) at room temperature (25°C) for about 10 minutes. This mixture was boiled for 1 hour using hot plate (Nour, Model 2007, Egypt).

(d) Microwave extraction (MIC)

Apple pomace powder (100 grams) was mixed with 2 liters of distilled water, and then, pH was adjusted to 1.9 using HCl (2 N). The extraction was performed using microwave oven (Samsung, Model MF245, Korea) at a power of 945 W for 30 minutes. This mixture was boiled for 1 hr using hot plate (Nour, Model 2007, Egypt).

(e) Ultrasound extraction (UL)

The extraction mixture is prepared by mixing 100 grams of apple pomace powder with 2 liters of water, the pH was adjusted to 1.9 by HCl (2 N), and then, it was sonicated for 30 minutes at 100% amplitude (20 kHz, maximum power of 700 W) using an ultrasonic device (Sonopuls HD 2070; Bandelin, Germany). This mixture was boiled for 1 hour using hot plate (Nour, Model 2007, Egypt).

**Step 2 (precipitation).**

All slurries were centrifuged using thermo centrifuge (THERMO, model MEGAFUGE 8 R, Germany) at 4000 rpm for 30 minutes (4°C). For the precipitation of pectin, 1 : 2 *V*/*V* ratio of ethanol was added and kept to stand for about 2 hours at room temperature, and then, it was centrifuged at 4000 rpm/20 minutes/4°C; the precipitated pectin was collected, then dried at 40°C in an oven (Shell Lab, Model 1370, California, USA), and kept at 4°C.

#### 2.2.2. Pectin Yield

It was calculated as

(1) $$\begin{array}{c} \text{Pectin yield}=\frac{\text{weight of pectin }\left(\text{M}0\right)}{\text{weight of pomace powder }\left(\text{M}\right)}\times 100. \end{array}$$

### 2.3. Characterization of Pectin

#### 2.3.1. Fourier Transform Infrared Spectroscopy (FTIR)

The structure analysis was examined by FT-IR 6000 spectrometer (JASCO, Japan from 400 to 4000 cm^−1^ wavenumber) [12].

#### 2.3.2. Degree of Esterification (DE) Using FTIR Spectrum

It was calculated by the following equation:

(2) $$\begin{array}{c} \text{DE }\left(\%\right)=\frac{\text{A}1740}{\text{A}1740+\text{A}1630}\times 100, \end{array}$$

where *A*1740 is the peak area at 1740 cm^−1^ (esterified groups) and *A*1630 is the peak area at 1630 cm^−1^ (carboxyl groups free) according to [12].

#### 2.3.3. Equivalent Weight (Eq.W)

The equivalent weight (Eq.W) of pectin samples was measured in triplicate as follows: pectin powder (0.5 gram) was completely dissolved in 100 mL of distilled water under continuous stirring (300 rpm) for 1 hr. 1 g of sodium chloride was added, followed by 5 drops of phenol red indicator, and the solution was titrated against 0.1 N NaOH until the color changed to pink and persisted for at least 30 seconds [13].

(3) $$\begin{array}{c} \text{Equivalent weigh}=\frac{\text{weight of sample }\left(\text{gm}\right)}{\text{volume of alkali }\left(\text{mL}\right)\times \text{normality of alkali}}\times 100. \end{array}$$

#### 2.3.4. Methoxyl Content (MC)

It was determined according to [13].

(4) $$\begin{array}{c} \text{Methoxyl content }\left(\%\right)=\frac{\text{volume of alkali }\left(\text{mL}\right)\times \text{normality of alkali}\times 3.1}{\text{weight of sample }\left(\text{G}\right)}. \end{array}$$

#### 2.3.5. Determination of Galacturonic Acid (GAI)

It was estimated according to [14].

(5) $$\begin{array}{c} \text{Galacturonic acid content }\left(\%\right)=\frac{176\times \text{methoxyl content }}{31\times \text{DM}}\times 100, \end{array}$$

where 176 and 31 are the molecular weight of galacturonic acid and methoxyl, respectively.

#### 2.3.6. Determination of Degree of Methylation (DM)

It was determined using the titration method according to [12].

(6) $$\begin{array}{c} \text{DM}=\frac{\text{V}2}{\text{V}2+\text{V}1}\times 100. \end{array}$$

#### 2.3.7. Scanning Electron Microscopy (SEM)

Morphological features of the particles were then observed by a field emission scanning electron microscope (Thermo Fisher Scientific, USA) at 6000.

#### 2.3.8. Zeta Potential

It was analyzed according to [15] using a dynamic light scattering method (Zetasizer Nano Zs, Malvern Instrument, Malvern, UK).

#### 2.3.9. Differential Scanning Calorimetry

Differential scanning calorimetry DSC131 evo (SETARAM Inc., France) was used, and the thermograms were processed using CALISTO (data processing software v.149) according to [16].

#### 2.3.10. X-Ray Diffraction Analysis (XRD)

An X-ray diffractometer (X'Pert3 Powder, PANalytical, Netherlands) was used to record XRD patterns, according to [17].

### 2.4. Antioxidant Activities

#### 2.4.1. DPPH Radical Scavenging Activity

The radical scavenging activity was estimated according to [18].

(7) $$\begin{array}{c} \text{Scavenging activity }\left(\%\right)=\frac{\text{AC}-\text{AS}}{\text{AC}}\times 100, \end{array}$$

where AC and AS are the absorbances at 517 nm of control and sample, respectively.

#### 2.4.2. Determination of Ferric Reducing Power (FRAP) Assay

Using FRAP assay according to [19], the results were expressed as l mole of Trolox equivalents/100 g of fresh weight sample.

### 2.5. Functional Properties

#### 2.5.1. Water and Oil Holding Capacity (WHC and OHC)

Pectin powder (1 g) was mixed into 10 mL of either distilled water or sunflower oil, and then, the obtained mixture was vortexed for 1 min and centrifuged for 30 min (at 3000×g). The supernatant was removed and the remnants were weighted [20].

#### 2.5.2. Emulsion Activity (EA) and Stability (ES)

Pectin aqueous solutions (0.5 and 2%) were prepared in the presence of 0.005% sodium azide. The emulsion activity (EA) and emulsion stability (ES) were measured according to [17] using the following equations:

(8) $$\begin{array}{c} \text{EA }\left(\%\right)=\frac{\text{VE}}{\text{VW}}\times 100,\\ \text{ES }\left(\%\right)=\frac{\text{VR}}{\text{VE}}\times 100, \end{array}$$

where VE is the volume of emulsified layer, VW is the volume of mixture, and VR is the volume of remaining emulsified layer after 30 days.

#### 2.5.3. Foaming Capacity (FC) and Stability (FS)

It was determined according to [21] with some modifications. Pectin solutions (0.5 and 2%) were prepared and then whipped by a homogenizer (CAT, Germany) at 7000 rpm/min for 3 min. Then, the pectin solutions were transferred into 50 mL cylinders. The foam volume was measured after 30 s and 30 min, respectively. Foaming capacity (FC) and stability (FS) were calculated as

(9) $$\begin{array}{c} \text{FC }\left(\%\right)=\frac{V1-V0}{V0}\times 100,\\ \text{FS }\left(\%\right)=\frac{V2-V0}{V0}\times 100, \end{array}$$

where *V*0 is the volume of mixture prior to whipping, *V*1 is the volume of mixture after 30 sec., and *V*2 is the volume of mixture after 30 min.

### 2.6. Statistical Analysis

The results were analyzed statistically using the SPSS program (version 20). The ANOVA test one way was used to analyze data. Data were represented as mean ± SD. Significance was considered at a level of 0.05.

## 3. Results and Discussions

There are many researches that have been conducted on the extraction of pectin from its different sources, using different methods of extraction. In this work, a comparison was made between the different extraction methods, whether the traditional method; green methods such as using microwave, ultrasound, and citric acid; and finally using a group of mixture together to improve the quality properties of the resulting pectin (citric acid, ascorbic acid, and acetic acid).

### 3.1. Extraction Yield

The yield of pectin extracted using five different extraction methods is shown in Table 1. It was varied from 14 to 22%. The results showed that extracting pectin using CI had a greater yield (22%) than other extraction methods. However, no significant differences between organic acid mixture and conventional extraction techniques were recorded. Organic acid mixture also has a lower dissociation constant than mineral acids (HCl); thus, their hydrolyzing capability is lower [22]. Also, Table 1 further demonstrates that MIC extraction yield was greater (17.6%) than both of HC and ORG extractions (14%); that finding was agreed with [23].

### 3.2. Characterization of Pectin

#### 3.2.1. FTIR

**Figure 1:**
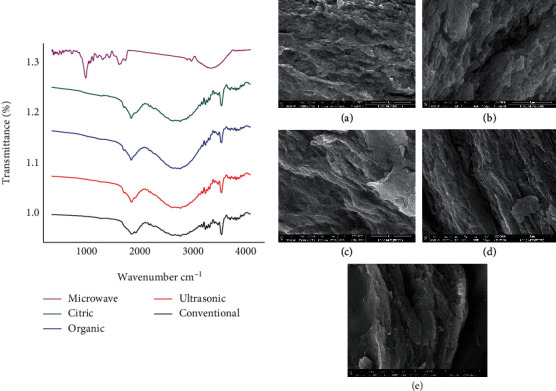
FT-IR spectrum and scanning electron microscopy (SEM) images of apple pectin extracted by CI (a), pectin extracted by HC (b), pectin extracted by UL (c), and organic acids (d) and pectin extracted by MIC (e).

The stretching of hydroxyl groups at approximately 3362 cm^−1^ and C–H stretching of CH2 groups at 2920 cm^−1^ were the main absorptions of hydroxyl groups. Except for the MIC sample, which had a shift in the OH group from 3362 to 3270.55 cm^−1^, the characteristic peaks for all pectin samples extracted by different methods were at 3362.6, 2920, 1730, and 1070 cm^−1^, corresponding to –OH, –CH, and C–O of acid and ester and –COC– stretching of the galacturonic acid, respectively (Figure 1). The C=O stretching vibration of ester carbonyl was identified at 1730 cm^−1^, whereas the C=O stretching vibration of carboxylate ion transmission was at 1637 cm^−1^. On the other hand, both MIC and UL samples showed high intensity, as shown in Figure 1; also, they exhibited higher peak areas linked to the degree of methylation and esterification, and the degree of methylation DM was determined using the ratio between these two peak areas (1743 cm^−1^ and 1637 cm^−1^) as shown in Equation (6). These findings were in line with those of [24]. In addition, both of MIC and UL exhibited high intensities at region 1200-1000 cm^−1^, which were located in the skeletal C–O and C–C vibration bands of glycosidic bonds and pyranoid rings and created a “finger print” region of carbohydrates; the position and intensity of the peaks are unique to a compound identification of the major functional groups [25]. The presence of pyranose was revealed by transmission peaks at 1015 cm^−1^ [23]. The extraction methods using CI, HC, and ORG had the lowest peak intensity at 1015 cm^−1^ and the lowest pyranose content. These results revealed that the pyranose was lost as a result of the extraction procedures. The findings also showed that both UL and MIC extraction methods may partially disrupt the covalent bonds between pectin and nonpectic polysaccharides. Sun and Tomkinson [26] discovered that ultrasound treatment may break the ether bonds and change the intensity and position of the peaks.

#### 3.2.2. Determination of Degree of Esterification (DE) by FTIR

It is associated with the extraction process and has a major impact on pectin's functional quality. The stronger bands at 1650 cm^−1^ and 1730 cm^−1^, which were free carboxyl groups and esterified groups, respectively, were linked to the peak intensities. Citric acid, ultrasonic, and traditional heat extraction did not appear to change its degree of esterification of apple pectin (63.42, 64.18, and 63.80 percent, respectively), which is consistent with prior research [27].

As a result of the increased peak intensity for ORG and MIC extraction, there was a little change in the DE of the extracted pectin when compared to the other extraction methods (64.55 and 64.80 percent, respectively) as shown in Table 1. Several studies found that using a high microwave power (945 W for 30 minutes) resulted in a greater DE of pectin. The breaking of the cell wall as a result of the microwave's high energy may have caused the increase in DE. During the microwave process and solubilization of pectin at low pH, hydrogen or ionic bonds are broken first, followed by covalent bond breaks (e.g., glycosidic linkages); the ester groups correlated with pectin can also be hydrolyzed by the acid (low pH) and high energy of the microwave [28].

#### 3.2.3. Equivalent Weight (Eq.W)

It is highly correlated with functional characteristics. The Eq.W of apple pectin ranged from 558 to 1158 g/mol (Table 1). Both of MIC and UL had a greater Eq.W (1158 g/mol). This finding was in agreement with [27]. This result might be attributed to the microwave array's influence on pectin degradation, which resulted in a smaller pectin particle remaining as a consequence of increased partial hydrolysis and partial degradation of pectin at higher temperatures and a longer extraction procedure.

#### 3.2.4. Methoxyl Content (MC)

As shown in Table 1, the methyl content of pectin ranged from 8.13 to 10.85%. Pectin extracted with ORG had the highest methyl content (10.85%). This might be because ORGS has lower hydrolyzing capabilities and has a lower dissociation constant. There was no significant difference (*p* > 0.05) between pectin extracted by CI and MIC (9.69 and 9.30%, respectively). This might be due to microwave heat or the low pH of the citric acid solution causing hydrolysis of methyl ester groups in the pectin structure [29].

#### 3.2.5. GAI Content

Determining GAI reflects pectin purity. As shown in Table 1, the GAI content of pectin extracted with HC was similar (no significant difference, *p* > 0.05) to the UL-extracted pectin sample (64.1 and 64.9 percent), whereas pectin extracted with CI and ORG had higher GAI contents, ranging from 87.58 to 85.82%, respectively. On the other hand, MIC resulted in a lower GAI concentration (68.53%) (Table 1). These results may be due to the lack of proteins, carbohydrates, and sugars in the precipitated pectin [30].

#### 3.2.6. Degree of Methylation (DM)

Pectins can be classified according to their degree of methylation (DM): high-methoxyl pectin (DM > 50%) and low-methoxyl pectin (DM < 50%). The degree of methylation (DM) was varied from 64% to 77%, so mostly, high-methoxyl pectin was extracted. Lower DM values resulted from extraction with citric acid method. The higher DM was for microwave extraction (77.02%). This result is similar to [12].

#### 3.2.7. Morphology of Extracted Pectin

As shown in Figure 1, SEM micrographs revealed that all pectin surfaces were unequal. SEM images of pectin samples extracted by HC and CI are shown in Figure 1. The surface morphology of the extracted pectin was altered by HC and CI extraction methods to a more porous surface structure with hollow holes. On the other hand, organic acid mixture-extracted pectin ORG and ultrasonic UL-extracted pectin proved to be different from the other samples CI and HC. The surfaces of the ORG and UL samples were more compact, multilaminated, and flaky and looked to be extremely hard (Figure 1). Rodsamran and Sothornvit [28] observed that apple pomace pectin has uneven and rough surfaces, which was in agreement with our findings.

#### 3.2.8. Zeta Potential of Pectin

All pectin samples (Figure S1) displayed negative zeta potential, although pectin extracted with CI had a lower value of zeta potential (-3.6 mV) than pectin extracted with the other methods, which varied from -29.42 to -59.42 mV. The organically extracted pectin was differentiated by having the greatest negative zeta potential (-59.42), followed by ultrasonically extracted pectin (-43). The significant negative charge showed that ORG pectin particles were more stable in aqueous dispersion than CI, UL, or HC methods. Particles having zeta potential values greater than or equal to ±30 mV is generally regarded to constitute a stable dispersion. Also, acidic polysaccharides such as pectin, with low zeta potentials (30 zeta potential > −30 mV), have a tendency to coagulate or flocculate, and it is difficult to have a stable colloidal or emulsion system at the electrokinetic potential, because a high zeta potential implies resistance to consistency or coalescence of emulsion [31].

#### 3.2.9. Differential Scanning Calorimetric (DSC)

There were two main peaks that were obtained during the thermal investigation of the pectin samples (Figure S2). The first peak was an endothermic peak between 50 and 150°C, which was linked to the evaporation of water molecules. The second peak, which occurred between 210 and 270°C, was exothermic pectin degradation. According to DSC curves, the endothermic peaks of pectin extracted by various methods were found to be at 123, 122, 116, 109, and 103° C for pectin extracted by HC, ORG, UL, MIC, and CI, respectively. The peak of pectin extracted by CI shifted slightly to the lower temperature. The greater water content and altered structure of pectin extracted by CI could explain this shift. Furthermore, pectin extracted with ORG had the lowest intensity of heat flow. More significant alterations in the ORG as an extractant for pectin samples were discovered, resulting in the pectin samples having more thermal stability [31]. At temperatures ranging from 253, 250, 243, 239, to 236°C, exothermic peaks were observed in pectin samples extracted with HC, ORG, UL, MIC, and CI, respectively. ORG extraction had a higher thermal stability of pectin than other extraction methods.

#### 3.2.10. X-Ray Diffraction (XRD)

It was employed to understand pectin structure (amorphous or crystalline). As shown in Figure S3, the X-ray diffractogram showed the differences between the extraction methods. As indicates in Table S4, the X-ray pattern showed distinct characteristic sharp peaks at equals 2*θ*. All pectin samples exhibited crystalline behavior. These findings were clarified by indicating that the XRD patterns of the pectin extraction methods did not differ, which revealed that the pectin crystallization had similar 2*θ* angles. However, at 2*θ* less than 20, there were distinctive peaks which gave the pectin an amorphous behavior. This demonstrated that pectin had a semicrystalline behavior in addition to mixture of two types of molecular network structures. As a result of the extraction methods, sharp peaks disappeared from MIC, HC, and ORG extractions. Taghizadeh and Abdollahi [32] demonstrated that it may be due to the decrement in the molecular weight of these pectin molecules.

### 3.3. Antioxidant Activity of Pectin Samples

**Figure 2:**
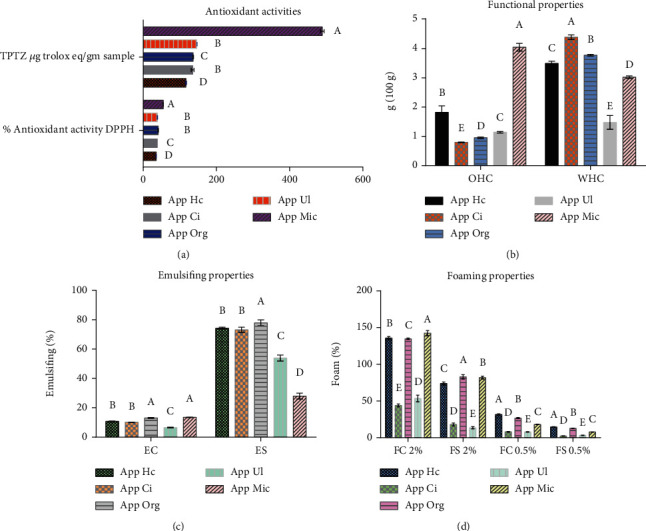
Antioxidant activities, water holding capacity WHC, oil holding capacity, emulsifying capacity (EC), emulsifying stability (ES), foaming capacity and stability, and OHC of pectin extracted with different methods. HC: heat conventional, CI: citric acid, ORG: organic acids, UL: ultrasonic; MIC: microwave. Values (mean ± SE) followed by different letters within the same exposure interval differ significantly (*p* < 0.05) (ANOVA) (Duncan test).

In accordance with the data shown in Figure 2, there was a significant difference between pectin extraction methods in DPPH radical scavenging activity. MIC pectin gave the highest significant (*p* < 0.05) DPPH radical scavenging activity followed by both of UL and ORG pectin. As can be seen in Figure 2, the reducing power activity method (FRAP) showed the same trend of DPPH method of antioxidant activity. MIC gave 3 to 4 times more antioxidant activity than other extraction methods (*p* < 0.05). The results of the antioxidant activity were closely correlated with the charts obtained from FTIR, at wavenumber 3000, which showed that the hydroxyl, NH, and free methyl groups had the largest peak for pectin extracted by MIC, followed by the extracted with ORG and extracted by UL. It is worth noting that the antioxidant activity was consistent with both degree of esterification and the degree of methylation; the higher the degree of esterification and methylation, the higher the antioxidant activity. Xiong et al. [33] suggested that the antioxidant activity of pectin might be due to the higher content of electrophilic groups that could accelerate the release of hydrogen from OH bonds and act as an electron donor to terminate the free radical chain reactions of rancidity. Also, this might be correlated to the fact that the hydroxyl groups' content was altered during the extraction treatment.

### 3.4. Functional Properties of Pectin Samples

#### 3.4.1. Water and Oil Holding Capacity

As shown in Figure 2(b), there were significant differences between the five methods of pectin extraction in both of water and oil holding capacity. Furthermore, the pectin extracted by CI gave the highest value of WHC followed by ORG method (4.4 and 3.5 grams water/gram, respectively). The MIC gave the lowest value. Moreover, the results of OHC had the opposite trend, where CI pectin gave the lowest oil holding capacity followed by ORG pectin (0.8 and 0.9 gram oil/gram, respectively), while MIC pectin was found to have the highest OHC (4 gram oil/gram sample), which could be attributed to its porosity, as seen from scanning electron microscopy images (see Figure 1) in addition to the high degree of methylation DM and esterification DE, which were related to FTIR charts as increasing in the intensities; the band area of esterified carboxyl groups at 3000 cm^−1^ indicated an increased DE. This result indicates that it would be particularly suitable for use as an ingredient for the stabilization of high-fat food products. The smaller polymer chain length as evident from particle size, DE, and GAI explained the lowest WHC of MIC pectin compared to CI- and ORG-extracted pectins.

It seems clear that the ability to retain water has been correlated with the physical and chemical properties of pectin, as previously explained, which showed that the higher value of MC and GAI, the more zeta potential charges, the great WHC properties. On the other side, the Eq.W has a reversed correlation with WHC which mean that pectin with low Eq.W has a high WHC value. Rubio-Senent et al. [34] suggested that pectin with high WHC and OHC could be used in some products such as cake and meat products to improve the quality of products.

#### 3.4.2. Emulsifying Properties

The emulsified capacity (EC) of MIC pectin was the highest value followed by ORG pectin (13.51 and 13.16%, respectively), while UL pectin had the lowest percentage of EC (6.5%). On the contrary, ORG pectin gave the highest emulsion stability, ES 78%, and MIC pectin gave a less stable emulsion 28%. These results confirm the possibility of using ORG-extracted pectin as an emulsifier and texture stabilizer in food processing (Figure 2(c)). Kazemi et al. [35] suggested that the small protein fractions that are linked to the pectin species may act as surfactant agents with emulsifying ability. These results confirm the possibility of using pectin extracted by organic acid mixture as an emulsifier and texture stabilizer in food processing.

#### 3.4.3. Foaming Properties

Both foaming capacity and stability are depending on the interfacial behaviors of the compounds with surface activity. The primary hydrophilic properties of the polysaccharides are the reason for their qualification to be used as foam thickeners and stabilizers.  Figure 2(d) shows the foaming properties of pectin extracted with different methods, at both concentrations of 0.5 and 2%. Generally, the higher the percentage of pectin, the better the foaming properties. MIC-, ORG-, and HC-extracted pectins had high closed foaming capacities (142.5, 136, and 135%, respectively) and foaming stability (82, 83, and 74%, respectively), while UL and CI had the lowest foaming capacity (53.5 and 44%) and stability (3.25 and 2.25). As shown in Figure 2(d). Bayar et al. [20] found the same results for FC and FS of *Opuntia ficus-indica* cladode pectin solutions.

## 4. Conclusion

Comparative study of different pectin extraction methods showed that there was an improvement of pectin properties for ORG, MIC, UL, and CI extraction methods. The results obtained demonstrated that both MIC and ORG extraction methods were efficient for good pectin physicochemical and functional properties and can be used in food production in high-temperature products, like those in the bakery industry (cakes, bread, and pastries).

## Figures and Tables

**Table 1:** Yield, degree of methylation, and degree of esterification for apple pectin extracted by different methods.

Sample	Yield extraction^∗^ (%)	Degree of methylation^∗^ (%)	Degree of esterification^∗^ (%)	Methyl content^∗^ (%)	GAI content^∗^ (%)	Eq. weight^∗^ (gm/mol)
Conventional method	14^a^	72.02^b^ ± 1.11	63.80	8.13^a^ ± 0.39	64.10^a^±3.9	1000^b^ ± 43.52
Citric acid	22^c^	64.05^a^ ± 0.95	63.42	9.69^c^ ± 0.39	85.82^b^±2.17	558.59^a^ ± 21.87
Organic acids	14^a^	70.25^b^ ± 0.25	64.55	10.85^d^ ± 0.05	87.58^b^±0.2	731.22^a^ ± 66.77
Microwave	17.6^b^	77.00^c^ ± 1	64.80	9.30^bc^ ± 0.78	68.53^a^±4.83	1158.44^b^ ± 167.27
Ultrasonic	16^b^	76.75^c^ ± 1.75	64.18	8.79^ab^ ± 0.23	64.90^a^±3.07	1158.44^b^ ± 167.27

^∗^Means in each column with different letters are significantly different (*p* < 0.05) ±SD (ANOVA) (Duncan test).

## Data Availability

The data that support the findings of this study are available from the corresponding author upon reasonable request.

## Abbreviations

WHC and OHC: Water and oil holding capacity
EA: Emulsion activity
ES: Emulsion stability
FC: Foaming capacity
FS: Foaming stability
HC: Heat conventional
ORG: Organic acid mixture
UL: Ultrasound extraction
MIC: Microwave extraction
DSC: Differential scanning calorimetry
XRD: X-ray diffraction analysis
FTIR: Fourier transform infrared
SEM: Scanning electron microscopy.

## Additional Points

*Highlights/Novelty*. (i) There is a comparative study between the nonconventional methods of pectin exaction: microwave, ultrasound, and citric and organic acid mixture. (ii) Organic aids and microwave were efficient for the extraction of pectin with high methyl content GAI and antioxidant activity. (iii) Also, high qualities of physicochemical and functional properties were achieved.

## Supplementary Materials

Supplementary Materials Figure S1: zeta potential (*ζ*) of apple pectin extracted by citric acid (CI), convention heat (HC), ultrasonic (UL), microwave (MIC), and organic acid (ORG) methods. Figure S2: DSC of apple pectin extracted by different methods. Figure S3: XRD of apple pectin extracted by different methods. Table S4: XRD diffractogram of pectin extracted from apple by different methods has been shown in Figures 4 (a, b, c, d, e and f).

## References

[1] Marić M., Grassino A. N., Zhu Z., Barba F. J., Brnčić M., Rimac Brnčić S. (2018). An overview of the traditional and innovative approaches for pectin extraction from plant food wastes and by-products: ultrasound-, microwaves-, and enzyme- assisted extraction. *Trends in Food Science & Technology*.

[2] Zhang F., Wang T., Wang X., Lü X. (2021). Apple pomace as a potential valuable resource for full-components utilization: a review. *Journal of Cleaner Production*.

[3] Tyagi V., Sharma P., Malviya R. (2015). Pectins and their role in food and pharmaceutical industry: a review. *Journal of Chronotherapy and Drug Delivery*.

[4] Zhu D., Zhang Y., Kou C., Xi P., Liu H. (2022). Ultrasonic and other sterilization methods on nutrition and flavor of cloudy apple juice. *Ultrasonics Sonochemistry*.

[5] Wang W., Ma X., Jiang P. (2016). Characterization of pectin from grapefruit peel: a comparison of ultrasound- assisted and conventional heating extractions. *Food Hydrocolloids*.

[6] Villamil-Galindo E., Piagentini A. M. (2022). Sequential ultrasound-assisted extraction of pectin and phenolic compounds for the valorisation of ‘Granny Smith’ apple peel. *Food Bioscience*.

[7] Cho E.-H., Jung H.-T., Lee B.-H., Kim H.-S., Rhee J.-K., Yoo S.-H. (2019). Green process development for apple-peel pectin production by organic acid extraction. *Carbohydrate Polymers*.

[8] Singla M., Sit N. (2021). Application of ultrasound in combination with other technologies in food processing: a review. *Ultrasonics Sonochemistry*.

[9] Swamy G. J., Muthukumarappan K. (2017). Optimization of continuous and intermittent microwave extraction of pectin from banana peels. *Food Chemistry*.

[10] Cui J., Zhao C., Feng L. (2021). Pectins from fruits: relationships between extraction methods, structural characteristics, and functional properties. *Trends in Food Science & Technology*.

[11] Mahmoud M. H., Abou-Arab A. A., Abu-Salem F. M. (2015). Effect of some different drying methods on the chemical analysis of citrus by-products. *Research Journal of Pharmaceutical Biological and Chemical Sciences*.

[12] Qin Z., Liu H.-M., Cheng X.-C., Wang X.-D. (2019). Effect of drying pretreatment methods on structure and properties of pectins extracted from Chinese quince fruit. *International Journal of Biological Macromolecules*.

[13] Ranganna S. (1986). *Handbook of Analysis and Quality Control for Fruit and Vegetable Products*.

[14] Xu S.-Y., Liu J.-P., Huang X. (2018). Ultrasonic-microwave assisted extraction, characterization and biological activity of pectin from jackfruit peel. *LWT*.

[15] Mahmoud M. H., Mehaya F. M., Abu-Salem F. M. (2020). Encapsulation of pomegranate seed oil using w/o/w nano-emulsion technique followed by spray drying and its application in jelly form. *Journal of Microbiology, Biotechnology and Food Sciences*.

[16] Azab D. E. S. H., Almoselhy R. I., Mahmoud M. H. (2022). Improving the quality characteristics of low fat toffee by using mango kernel fat, pectin, and high-speed homogenizer. *Journal of Food Processing and Preservation*.

[17] Azab D. E. H., Heika Y. A., Salaheldin T. A., Hassan A. A., Abu-Salem F. M. (2019). Nano formulated soy proteins for improvement of beef burgers quality. *Egyptian Journal of Chemistry*.

[18] Aboelsoued D., Abo-Aziza F. A. M., Mahmoud M. H., Abdel Megeed K. N., Abu el Ezz N. M. T., Abu-Salem F. M. (2019). Anticryptosporidial effect of pomegranate peels water extract in experimentally infected mice with special reference to some biochemical parameters and antioxidant activity. *Journal of Parasitic Diseases*.

[19] de Moraes Barros H. R., de Castro Ferreira T. A., Genovese M. I. (2012). Antioxidant capacity and mineral content of pulp and peel from commercial cultivars of citrus from Brazil. *Food Chemistry*.

[20] Bayar N., Bouallegue T., Achour M., Kriaa M., Bougatef A., Kammoun R. (2017). Ultrasonic extraction of pectin from *Opuntia ficus indica* cladodes after mucilage removal: optimization of experimental conditions and evaluation of chemical and functional properties. *Food Chemistry*.

[21] Hassan A. B., Mahmoud N. S., Elmamoun K., Adiamo O. Q., Mohamed Ahmed I. A. (2018). Effects of gamma irradiation on the protein characteristics and functional properties of sesame (*Sesamum indicum* L.) seeds. *Radiation Physics and Chemistry*.

[22] Kermani Z. J., Shpigelman A., Kyomugasho C. (2014). The impact of extraction with a chelating agent under acidic conditions on the cell wall polymers of mango peel. *Food Chemistry*.

[23] Dranca F., Vargas M., Oroian M. (2020). Physicochemical properties of pectin from *Malus domestica* ‘Fălticeni’ apple pomace as affected by non-conventional extraction techniques. *Food Hydrocolloids*.

[24] Pappas C. S., Malovikova A., Hromadkova Z., Tarantilis P. A., Ebringerova A., Polissiou M. G. (2004). Determination of the degree of esterification of pectinates with decyl and benzyl ester groups by diffuse reflectance infrared Fourier transform spectroscopy (DRIFTS) and curve-fitting deconvolution method. *Carbohydrate Polymers*.

[25] Urias-Orona V., Rascón-Chu A., Lizardi-Mendoza J., Carvajal-Millán E., Gardea A. A., Ramírez-Wong B. (2010). A novel pectin material: extraction, characterization and gelling properties. *International Journal of Molecular Sciences*.

[26] Sun R., Tomkinson J. (2002). Comparative study of lignins isolated by alkali and ultrasound-assisted alkali extractions from wheat straw. *Ultrasonics Sonochemistry*.

[27] Guandalini B. B. V., Rodrigues N. P., Marczak L. D. F. (2019). Sequential extraction of phenolics and pectin from mango peel assisted by ultrasound. *Food Research International*.

[28] Rodsamran P., Sothornvit R. (2019). Microwave heating extraction of pectin from lime peel: characterization and properties compared with the conventional heating method. *Food Chemistry*.

[29] Picot-Allain M. C., Nancy B. R., Emmambux M. N. (2022). Extraction, characterisation, and application of pectin from tropical and sub-tropical fruits: a review. *Food Reviews International*.

[30] Ismail N. S. M., Ramli N., Hani N. M., Meon Z. (2012). Extraction and characterization of pectin from dragon fruit (*Hylocereus polyrhizus*) using various extraction conditions. *Sains Malaysiana*.

[31] Misra N. N., Yadav S. K. (2020). Extraction of pectin from black carrot pomace using intermittent microwave, ultrasound and conventional heating: kinetics, characterization and process economics. *Food Hydrocolloids*.

[32] Taghizadeh M. T., Abdollahi R. (2011). Sonolytic, sonocatalytic and sonophotocatalytic degradation of chitosan in the presence of TiO_2_ nanoparticles. *Ultrasonics Sonochemistry*.

[33] Xiong B., Zhang W., Wu Z. (2021). Preparation, characterization, antioxidant and anti-inflammatory activities of acid-soluble pectin from okra (*Abelmoschus esculentus* L.). *International Journal of Biological Macromolecules*.

[34] Rubio-Senent F., Rodríguez-Gutiérrez G., Lama-Muñoz A., Fernández-Bolaños J. (2015). Pectin extracted from thermally treated olive oil by-products: characterization, physico-chemical properties, *in vitro* bile acid and glucose binding. *Food Hydrocolloids*.

[35] Kazemi M., Khodaiyan F., Hosseini S. S. (2019). Utilization of food processing wastes of eggplant as a high potential pectin source and characterization of extracted pectin. *Food Chemistry*.

