# Supplementary material for: A Comparative Study of Pectin Green Extraction Methods from Apple Waste: Characterization and Functional Properties
Source: Int J Food Sci. 2022 Dec 19;2022:2865921. doi: 10.1155/2022/2865921 (PMC9792233; doi:10.1155/2022/2865921)
Supplement: Supplementary Materials — Figure S1: zeta potential (ζ) of apple pectin extracted by citric acid (CI), convention heat (HC), ultrasonic (UL), microwave (MIC), and organic acid (ORG) methods. Figure S2: DSC of apple pectin extracted by different methods. Figure S3: XRD of apple pectin extracted by different methods. Table S4: XRD diffractogram of pectin extracted from apple by different methods has been shown in Figures 4 (a, b, c, d, e and f). [file 2865921.f1.docx]

**Supplementary**

**Fig. S1.** Zeta potential (ζ) of apple pectin extracted by citric acid (CI) convention heat (HC),Ultrasonic (UL), Microwave ( MIC) and organic acid (ORG) methods.

App HC

App CI

App ORG

App Ul

App MIC

**Fig. S2.** DSC of apple pectin extracted by different methods.


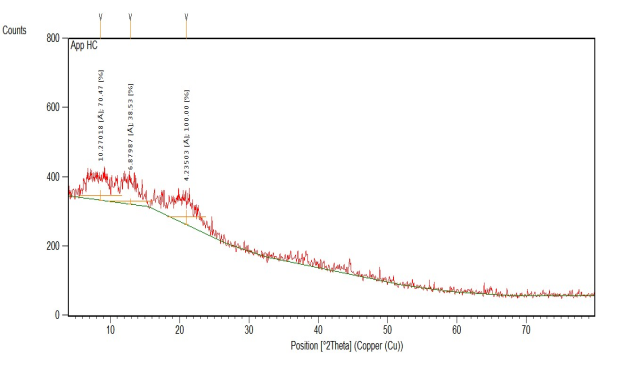

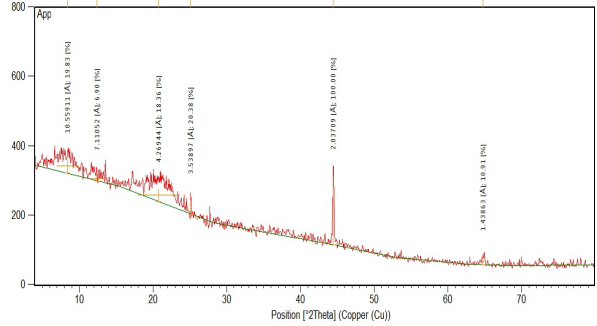

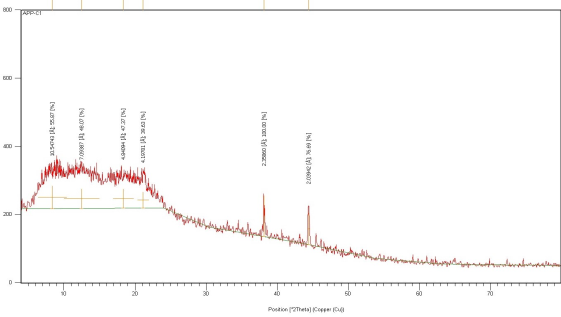

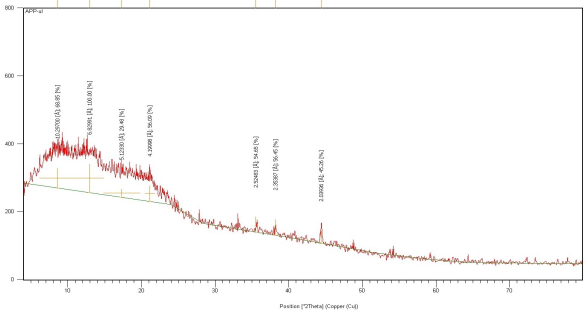

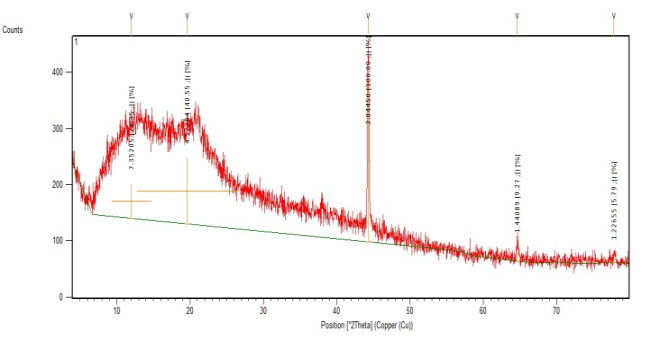


a

b

c

d

e

**Fig. S3.** XRD of apple pectin extracted by different methods.

**Table. S4** The XRD diffractogram of pectin extracted from apple by different methods have been shown in Fig. 4 (a, b, c, d, e and f).

| samples |  |  | 2 θ |  |  |  |
| --- | --- | --- | --- | --- | --- | --- |
| CI | 8.4 | 12.5 | 18.3 | 21.15 | 38.12 | 44.38 |
| HC | 9 | 13 | - | 21 | - | 44 |
| MIC | - | 12.16 | 19.9 | 32.8 | 38 | 44.26 |
| UL | 8.6 | 13 | 17 | 21.14 | 35.5 | 44.3 |
| ORG | 8.4 | 12.4 | 21 | 25.1 | - | 44.43 |
